# Long term outcome of acute kidney injury due to leptospirosis? A longitudinal study in Sri Lanka

**DOI:** 10.1186/1756-0500-7-398

**Published:** 2014-06-25

**Authors:** Nalaka J Herath, Senanayake AM Kularatne, Kosala GAD Weerakoon, Abdul Wazil, Nilakshi Subasinghe, Neelakanthi VI Ratnatunga

**Affiliations:** 1 Nephrology and Transplant unit, Teaching Hospital, Anuradhapura, Sri Lanka; 2 Department of Medicine, Faculty of Medicine, University of Peradeniya, Peradeniya, Sri Lanka; 3 Department of Parasitology, Faculty of Medicine and Allied Sciences, Rajarata University of Sri Lanka, Saliyapura, Anuradhapura, Sri Lanka; 4 Nephrology and Transplant unit, Teaching Hospital, Kandy, Sri Lanka; 5 Department of Pathology, Faculty of Medicine, University of Peradeniya, Peradeniya, Sri Lanka

**Keywords:** Leptospirosis, Acute kidney injury, Chronic kidney disease, Long term renal outcome, Sri Lanka

## Abstract

**Background:**

Leptospirosis is an important zoonotic disease of variable severity and is a common cause of acute kidney injury (AKI) in tropics. However the knowledge on long term renal outcome in leptospirosis is scarce. This study aims to assess the long-term renal outcome of AKI caused by leptospirosis.

**Findings:**

Hospital records of patients who had developed AKI following leptospirosis (Serologically confirmed) presented to two Teaching Hospitals in Kandy district over 3 years from 2007 were studied. A total of 44 patients were included and they had been followed up at least for one year in out patient clinics with regular assessment including renal status. Renal histology was studied in two patients. The primary outcome measure was normalization of renal function at one year. Of the 44 patients, 31 were in the risk and injury stage (Group 1), and the rest of them were in the failure stage (Group 2) under RIFLE criteria. Of group 2 patients, 11 had abnormal renal functions on discharge. Their mean serum creatinine and GFR values on discharge were 392 mmol/l and 20 ml/min/1.73 m^2^. Other two patients had full renal recovery whilst in the hospital. Nine in the group 2 required renal replacement therapy by means of peritoneal dialysis, intermittent haemodialysis or haemofiltration. Seventeen out of the total had persistently abnormal renal functions on discharge. Of them 13 recovered their renal functions to normal. Four patients (9%) who belonged to group 2, had persistently abnormal renal functions after first year compatible with stage 3 chronic kidney disease (CKD). Renal histology of two patients showed tubulointerstitial lymphocyte infiltrate, tubular atrophy and interstitial fibrosis.

**Conclusion:**

The long term renal outcome of AKI following leptospirosis is satisfactory as only 9% of patients had abnormal renal functions compatible with early stage of CKD. Even among them, advanced CKD or dialysis dependency had not been observed.

## Findings

### Background

Leptospirosis is a globally prevalent important zoonosis caused by pathogenic strain of *Leptospira* an aerobic spirochete. Transmission of the disease to human occurs usually through contact with water or soil contaminated by urine of larger array of infected animals [1,2]. Leptospirosis has become an important public health problem in Sri Lanka, a country which has agriculture based economy where farmers have become the common victim of the disease. Major outbreaks of leptospirosis were frequent in different parts of the island and the year 2008 showed one of the worst outbreaks in the Central Province of Sri Lanka in which many pathogenic serovars were identified [3]. In this outbreak, a single tertiary care hospital alone managed 227 cases, of them 9 patients developed severe acute kidney injury (AKI) who needed dialysis support [4]. The incidence of AKI in leptospirosis varies from 10% to 60%, depending on multiple factors such as age, severity of the disease and the causative serovars of a region [5].

The renal involvement in leptospirosis can vary from a subclinical course, with mild proteinuria and abnormal urinary sediments to an overt AKI. The AKI usually presents with a rapid elevation in blood urea and creatinine, and can be associated with jaundice. Kidney injury in patients with hyperbilirubinaemia represents a severe form of the disease frequently accompanied by oliguria or anuria [5]. On the contrary, AKI also presents in the non-oliguric form with hypokalaemia in 41% to 45% of the patients with leptospirosis [6]. Renal impairment is a frequent complication in severe disease mainly characterized by interstitial and tubular damage [7]. The contributory factors for development of AKI could be the acute infection of the kidney with the pathogen or an immune mediated renal damage. In addition, haemodynamic alterations, hyperbilirubinaemia and rhabdomyolysis are also associated with the genesis of AKI in leptospirosis [6,7]. The outcome of AKI if managed properly is fair, but a significant proportion could succumb to it [4]. There are hardly any follow up data of long term renal outcome in leptospirosis in the literature. This study aims to assess the long-term renal outcome of AKI caused by leptospirosis.

## Methods

### Study setting and sample selection

The study was carried out at two Teaching Hospitals in Kandy district of Sri Lanka (Peradeniya and Kandy). Hospital records of patients who had developed AKI following leptospirosis during the period of 2007 to 2010 were studied. These patients had been followed up at least for one year in the out patient clinics with regular assessment including renal status. The relevant clinical data and investigation results were collected from the hospital records. Further clinical assessments of the patients were done at the outpatient clinics and necessary additional details were documented. Moreover, we were able to retrieve renal histology reports of two patients who had undergone renal biopsies 6 month after the initial infection due to persistently abnormal renal functions.

This study is a part of the leptospirosis studies we are carrying out, for which the ethical approval has been obtained from the Ethics Committee, Faculty of Medicine, University of Peradeniya, Sri Lanka. Further we have obtained permission from the two hospitals to (Teaching Hospital, Peradeniya and Teaching Hospital, Kandy, Sri Lanka) to conduct this study in these hospitals.

### Definitions

We classified patients according to the maximum RIFLE criteria (named by the severity of renal impairment: Risk, Injury, Failure, Loss, End-stage kidney disease) [8] reached during their hospital stay.

CKD was defined as renal structural abnormalities in imaging studies (size, echogenicity and loss of cortico-medullary demarcations), continued presence of abnormal urine test results (presence of protein, cells and casts) and glomerular filtration rate (GFR) <60 ml/ min/1.73 m^2^ lasting more than 3 months. The GFR was calculated by using an equation derived from the Modification of Diet in Renal Disease Study [9].

According to our unit guidelines for renal replacement therapy (RRT) in AKI, peritoneal dialysis is the initial mode of RRT in all patients who required it, unless contraindicated. If the patient is dialysis dependant beyond day four , the next mode of RRT is haemodialysis. Haemofiltration is the desired mode of RRT, whenever a patient is haemodynamically unstable with contra-indication for peritoneal dialysis.

There were 44 patients who had AKI due to leptospirosis. Laboratory confirmation of leptospirosis was done with serology. However serovar identification was not done in all the patients and only a limited number of identifications were done [3]. The group one consisted of 31 patients who fulfilled risk and injury stage of RIFLE criteria whilst group two consisted 13 patients who had maximum serum creatinine level reached during their hospital stay of more than 355 mmol/l with a rise of >44 mmol/l. Therefore, the group 2 was considered in the failure stage under RIFLE criteria.

### Data collection

The age, gender, occupation, co-morbidities such as hypertension, diabetes, abuse of alcohol and past history of renal disease were taken from the data base. Further investigations such as urine full report, renal function tests, Ultra sound scan of Kidney, Ureter and Bladder (USS KUB), type and duration of RRT, indication for RRT and periodic serum creatinine levels during follow up were also taken from the database. Those who had pre-existing CKD were excluded. The primary outcome measure was the normalization of renal function at one year. Data were double-checked, entered into a database and analysed using SPSS version 12. The descriptive data are presented as proportions, mean, SD and range.

## Results

Out of 44 patients who were selected for the study, 36 (82%) were men and the mean age of the group was 43 years (range 21-69 years;SD,15). Total duration of hospital stay of these patients was 10 days (SD,4) and mean durations of the key clinical characteristics and laboratory investigation findings of the total group are summarized in Table 1. In Group 1 (n, 31) mean age was 44 years (range, 21–69 years; SD,14) while 27 (87%) were men. Of them, 25 normalised renal functions whilst in the hospital. At the end of one year, all patients in group one recovered their renal functions to normal. In group 2 (n,13) mean age was 42 years (range 19–67, SD – 17) and 9 (69%) were men. Of them, 11 patients had high serum creatinine at discharge from the hospital (Their mean serum creatinine and GFR values on discharge were 392 mmol/l (Range 86 – 588 mmol/l) and 20 ml/min/1.73 m^2^) while two patients had full renal recovery. In this group, 9 patients required peritoneal dialysis and 4 patients needed additional intermittent haemodialysis. One patient underwent haemofiltration followed by haemodialysis. During the follow up period, 7 patients regained normal renal functions, but 4 patients showed residual renal impairment compatible with stage 3 CKD. Mean serum creatinine values of these 4 patients on discharge and at one year were 574 mmol/l (Range: 327 – 930) and 130 (Range: 119–137) respectively with corresponding GFR values as 12 (Range: 7–18) and 54 ml/min/1.73 m^2^ (50–59). Thus, 9% of patients of this cohort had developed CKD. Kidney biopsies were done in two patients who had persistently abnormal urine and renal functions after six months. These biopsies showed mild patchy interstitial lymphocyte infiltrate, tubular atrophy and interstitial fibrosis with negative immunofluorescence and without any evidence of glomerular or vascular involvement suggestive of coexisting primary renal disease.

**Table 1:** Main clinical features and laboratory investigation findings of the total group during the hospital stay

**Description**	**Total duration in days (Mean, SD)**
**Clinical features with total duration in days**	
Fever	6 (2)
Headache	6 (2)
Vomiting	3 (2)
Conj. Injection	6 (3)
Abdominal pain	5 (2)
Dark coloured urine	3 (2)
***Basic laboratory investigation findings***	
Haemoglobin (g/dl)	12 (2)
Platelet count (x 106/l)	104 (57)
Haematocrit (%)	35 (7)
Leucocyte count (x 106/l)	10 (6)
Alanine amino transferase (U/l)	68 (42)
Aspartate amino transferase (U/l)	76 (48)
Total bilirubin (μmol/l)	38 (27)
Blood urea (mg/dl)	24 (11)
Serum creatinine (mmol/l)	306 (209)

## Discussion

This study found normalization of renal functions in 40 patients (91%) during the course of one year follow up following AKI due to leptospirosis. Twenty seven patients (61%) recovered their renal functions before the discharge from the hospital and all patients in group one had recovered their renal functions to normal during follow up. This indicates renal recovery is speedy and complete in mild to moderate AKI once underline infection is treated with appropriate antibiotics and with other life supportive measures such as correction of dehydration. In group two, nine recovered their renal functions to normal. Only 4 patients (9%) ended up with residual renal impairment compatible with early CKD.

As we were unaware of pre-morbid renal functions of this series of patients, there is a small possibility of having preexisting CKD in four patients who developed early CKD after one year of follow up. But we tried to minimize this compounding factor by excluding all patients who had any renal disease in the past or ultrasonic evidence of CKD performed during acute stage. One interesting finding was that all four who have got abnormal renal functions compatible with early CKD, none developed advanced CKD by end of one year. In contrast, in a cohort of AKI due to snakebite, a significant portion of patients developed either advanced CKD or dialysis dependency by the end of one year [10]. This difference of severity can be explained by the nature of renal pathology related to a specific etiology. Acute tubular necrosis (ATN) and acute tubulointerstitial nephritis were reported to be the most characteristic renal lesion observed in acute stage of leptospirosis [11]. Though we do not have renal histology during acute stage, we assume most of our patients had same pathology. High rate of complete renal recovery from AKI, absence of patients with advanced CKD or dialysis dependency at one year and follow up renal histology, support the established renal histopathology in leptospirosis. Most of our patients recovered which indicate reversibility of ATN. By contrast, AKI in snakebite has caused more irreversible glomerular necrosis and vascular damage which lead to dialysis dependency or advanced CKD early in the course.

Pathogenesis of CKD in leptospirosis is yet to be defined as there is a lack of longitudinal studies. This study suggests, ongoing chronic tubulointertitial nephritis may be a cause of early CKD. It is not clear whether persistence of initial renal insult or chronic leptospiral infection cause this ongoing chronic tubulointerstitial disease. Further research is needed to focus on this area to explore the second possibility.

## Conclusion

We studied the long-term renal outcome of AKI caused by leptospirosis. Nine percent of patients in this series of 44 patients showed abnormal renal functions compatible with early stage of CKD during the follow up. However, advanced CKD or dialysis dependency was not observed in this cohort of patients. Further research is needed to confirm the pathogenesis of CKD in post leptospirosis AKI.

## Competing interests

The authors declare that they have no competing interests.

## Authors’ contributions

SAMK and NJH conceived and designed the study. AW, NS and NJH collected data. KGADW contributed to data entry and analysis. NVIR did histopathology. SAMK, NJH and KGADW drafted the report. All authors contributed to review and revision of the report and have seen and approved the final version. SAMK is the guarantor.
